# Dissecting quantitative resistance to *Xanthomonas campestris* pv. *campestris* in leaves of *Brassica oleracea* by QTL analysis

**DOI:** 10.1038/s41598-019-38527-5

**Published:** 2019-02-14

**Authors:** Laura Iglesias-Bernabé, Pari Madloo, Víctor Manuel Rodríguez, Marta Francisco, Pilar Soengas

**Affiliations:** 1 Group of Genetics, Breeding and Biochemistry of Brassicas, Misión Biológica de Galicia (MBG-CSIC), Pontevedra, Spain; 2 University of Santiago de Compostela, A Coruña, Spain

## Abstract

Black rot, caused by the bacterium *Xanthomonas campestris* pv. *campestris* (*Xcc*), produces important economic losses in crops of *Brassica oleracea* worldwide. Resistance to race 1, the most virulent and widespread in *B*. *oleracea*, is under quantitative control. Knowledge about the genetics of this resistance would help in designing strategies to control initial stages of invasion and development of the disease. QTL analysis of the resistance in the BolTBDH mapping population was performed. Resistance was measured with five traits related to initial stages of the invasion, success of infection and spread of the pathogen. Four single-trait QTLs of resistance were found, from which one represent novel variation. After performing multi-trait QTL, we concluded that spread of *Xcc* is related to the size of the leaf. Individuals from the mapping population follow two different strategies to cope with the spread of the disease: reducing lesion size or maintain more area of the leaf photosynthetically active, being more tolerant to *Xcc* invasion. Mechanisms underlying variation for resistance may be related to different aspects of plant immunity, including the synthesis of glucosinolates and phenolics.

## Introduction

The species *Brassica oleracea* included many well-known crops such as kale, cabbage, broccoli, Brussels sprouts and cauliflower, grown and consumed worldwide. Black rot is one of the most important diseases that affect *B*. *oleracea* crops, causing great economic losses, reducing the performance and the quality of crops^1^. This disease is caused by the gram-negative bacterium *Xanthomonas campestris* pv. *campestris* (*Xcc*) (Pammel) Dowson. The pathogen enters the margins of the leaves across the hydathodes, or through wounds of the plant. Once inside, the pathogen travels through the vascular system, invading the xylem and colonizing the mesophyll. Typical symptoms include V-shaped chlorosis from the edges of the leaves, necrosis and darkening of veins of the leaves and of the vascular tissue of the stem. As the disease progresses, wilting and necrosis throughout the plant are produced^2^. Nowadays there is no effective method to control *Xcc*. Attempts are directed to employ agronomic practices, for example, by using planting material free of *Xcc* (seeds or transplants), eliminating other possible sources of inoculum such as residues of infected crops and cruciferous weeds, and employing rotary crops^3^. The use of potentially resistant *Xcc* crops is a measure to be adopted within an integrated management control. The search of sources of resistance is complicated due to the existence of nine races of the pathogen. Initially^4^, identified six races (1–6), and later^5^ added three additional ones (7–9). Race identification was based on avirulence/virulence patterns to six differential host genotypes, involving five resistance genes in the plant and five avirulence genes in the pathogen. Worldwide, race 1 is one of the most virulent and widespread races in *B*. *oleracea* crops, accounting for more than 90% of black rot disease around the world together with race 4^4^. Following extensive screening of *B*. *oleracea* accessions^3^, concluded that resistance to races 1 and 4 was either non-existent or very rare, whereas resistance to less important races (2, 3 and 6) was frequently found.

Dominant R-gene-mediated effector- triggered immunity (ETI) is considered to be the most efficient form of resistance in plants^6^. R genes encode proteins by recognize directly or indirectly corresponding effectors, mostly followed by hypersensitive response^7^. According to^3,4^ a R gene conferring resistance to race 1 (R1) is present in the B genomes of *B*. *carinata* (BBCC), *B*. *juncea* (AABB) and *B*. *nigra* (BB). However, this gene is absent in *B*. *oleracea* (CC). Although resistance of cauliflower line SN455 has been reported to be determined by a recessive allele of a single gene^8^, black rot resistance of most *B*. *oleracea* lines is considered to be under quantitative control^3,9^. Because ETI fails to provide durable and broad-spectrum resistance, increasing attention has been focused to quantitative resistance. Few R genes are responsible for quantitative disease resistance. On the contrary, identified genes accounting for quantitative resistance represent a broad range of molecular functions, including, for example, kinases and ABC transporters^6^.

Secondary metabolites can also be involved in quantitative resistance. *B*. *oleracea* is known for its characteristic and high content of glucosinolates (GSLs) and phenolic compounds. Upon cellular disruption, GSLs are hydrolyzed to various bioactive breakdown products, by the endogenous enzymes myrosinases, which have been proved to be toxic to pathogens. The accumulation of GSLs in transgenic Arabidopsis plants enhanced resistance to bacteria^10^. Besides GSLs may play a role in plant defense against *Xcc* in *B*. *oleracea*^11^. There are many works which describe the potent role of phenolic compounds in disease resistance^12,13^. Methanolic extracts from *B*. *rapa*, containing GSLs and phenolic compounds, inhibited the growth of *Xcc*^14^. The genetic and metabolic basis of phenolics and GSLs accumulation was dissected through analysis of total phenolics concentration and its individual components in the BolTBDH doubled haploid mapping population of *B*. *oleracea*. QTLs that had an effect on phenolics and GSLs concentration were integrated, resulting in 33 consensus QTLs controlling phenolics traits and 18 QTLs controlling GSLs^15,16^.

We propose to perform a QTLs analysis of the resistance to *Xcc* in BolTBDH population and to study the relationship between variation in disease severity and variation in the content of secondary metabolites. The role of possible candidate genes underlying variation for *Xcc* resistance is discussed.

## Materials and Methods

### Plant material and inoculation

The double haploid (DH) mapping population employed in this study (BolTBDH) was created from an F_1_ individual, derived by crossing a DH broccoli line ‘Early Big’ (P2) and a DH rapid cycling of Chinese kale line (TO1000DH3, P1)^17^. Parents and 137 DH lines were grown in greenhouse under controlled conditions: a photoperiod of 14 h light, temperature 18 °C at night and 25 °C during the day, and a relative humidity of 50%. Plants were sown in a completely randomized experiment with two replications and four plants per replication. A resistance test was performed with race 1 of *Xcc*. Race 1 type strain HRI3811 was provided by Joana Vicente (University of Warwick, UK). Bacterial cultures were grown in PDB (Sigma-Aldrich, St. Louis, USA) at 30 °C for 48 h. Bacterial cultures were diluted in sterile distilled water. Turbidity of the suspension was measured with a microplate spectrophotometer (Spectra MR; DynexTechnologies, Chantilly, VA) at a wavelength of 600 nm. The suspension was diluted to reach an absorbance of 0.5, which corresponds to a concentration of 5 × 10^8^cfu/ml.

Two consecutive inoculations were made in the same plants for testing the resistance. The first inoculation was carried out eight weeks after sowing. Mouse tooth forceps wrapped in cotton submerged in the inoculum were used for inoculation biting on three veins located on the edge of the leaf. One leaf per plant was inoculated. After inoculation, greenhouse conditions were set to 14 h light, temperature 24 °C at night and 28 °C during the day (with an oscillation of ±2 °C) and relative humidity above 80%. The inoculated leaves were collected two weeks after inoculation. Five days afterwards, the same procedure of inoculation and harvesting of inoculated leaves was repeated in the same plants (the second inoculation).

### Resistance traits

Five traits were recorded in both inoculations as measures of resistance: initial index (INDEX0), final index (INDEXF), diseased leaf area (DISAREA), percentage of diseased leaf area (%DISAREA) with respect to total leaf area (TOTAREA), and percentage of points showing symptoms (%DISPOINTS). Subjective scores were assessed on a visual 1 to 9 rating scale based on the relative lesion size, 6 (INDEX0) and 14 (INDEXF) days after each inoculation, where 1 = no visible symptoms and 9 = severely diseased with typical V- shaped chlorotic leaf edge lesions presenting blackened veins areas^18^. One single measure was made for each leaf then McKinney index^19^ was computed by repetition. Pictures of harvested leaves were taken 14 days after the first and the second inoculations. DISAREA and TOTAREA were calculated with the software ImageJ 1.50i (Wayne Rasband, National Institutes of Health, USA) using the photos of leaves from both harvests. Then %DISAREA was computed for each leaf individually by dividing DISAREA into TOTAREA and multiplying by 100. Means of DISAREA, TOTAREA and %DISAREA were calculated by line and repetition. Finally %DISPOINTS was computed as the percentage of points which showed symptoms in the whole of the leaves of a repetition over the total amount of inoculated points in the same repetition.

### Statistical analysis

Analyses of variance were performed for disease traits. Genotypes, repetitions and inoculations were considered as sources of variation with PROC GLM of SAS^20^. Genotypes and inoculations were considered as fixed effects whereas replications were considered as a random factor. Means of parents were compared with a Student’s t at the 0.05 level of probability. Pearson’s correlation coefficients were computed between resistant traits with PROC CORR of SAS^20^.

The genetic map employed for the QTL analysis was created by Iniguez -Luy *et al*.^17^. It has 279 markers (SSRs and RFLPs) distributed along nine linkage groups assigned to chromosomes C1–C9 of *B*. *oleracea* with a total distance of 891.4 cM and a marker density of 3.2 cM/marker. Single-trait QTL mapping was carried out through a composite interval mapping method (CIM)^21^ by using the software PLABMQTL^22^. Individual analyses were carried out for each trait and inoculation. A logarithm of odds (LOD) threshold was chosen for each trait in order to declare the putative QTL significant by following a permutation test, with N = 1000, and a critical alpha value of 10%^23^. The analysis and cofactor election were carried out by following PLABMQTL’s recommendations. The proportion of phenotypic variance explained for a specific trait was determined by the adjusted coefficient of determination of regression (R^2^) fitting a model which includes all detected QTLs. R^2^ were adjusted for the number of explanatory terms relative to the number of data point.

Multi-trait QTL analysis was carried out with the statistical Genstat version 19.1.0.2139. The variance-covariance model for QTL selection and fitting the QTL model was chose as “unstructured” based on the Bayesian information criterion (BIC). The threshold for QTL detection was calculated with a genome wide significance of p < 0.05^24^. QTL mapping was implemented in a stepwise manner starting with simple interval mapping followed by three rounds of CIM. Backward selection was conducted on QTL detected and the final effects were estimated based on multi-QTL model. Two different hypothesis were tested: existence of multi-trait QTLs for *Xcc* resistance and leaf size, and existence of multi-trait QTLs for *Xcc* resistance and content in phenolics and GSLs. Multi-trait QTL analysis was performed combining traits from both inoculations.

To identify possible candidate genes underlying variation for *Xcc* resistance we performed a GO analysis. Collinear genomic blocks between the BolTBDH mapping population and *A*. *thaliana* were identified by^17^. Genes of *A*. *thaliana* located in syntenic regions to those of resistant QTLs in *B*. *oleracea* were obtained from TAIR database. Then, GO enrichment analysis using the tool “Singular Enrichment Analysis” (SEA) from AgriGO was performed (http://bioinfo.cau.edu.cn/agriGO/) using the *A*. *thaliana* gene model (TAIR9) as background. A Fisher test with the Yekutielli multi-test correction method (significance threshold = 0.05) was used.

## Results

Individual analysis of data by inoculation was done, since the interaction genotype × inoculation was significant in the analysis of variance (data not shown). Correlations between INDEX0 and %DISPOINTS with the other traits were low or no significant, whereas INDEXF, DISAREA and %DISAREA showed correlations above 0.576 between them (Table 1).

**Table 1:** Pearson’s correlation coefficients between resistant traits and leaf area in the BolTBDH mapping population.

Traits	INDEX0	INDEXF	DISAREA	%DISAREA	%DISPOINTS	TOTAREA
INDEX0	1.000	0.0187*	0.044	−0.025	0.105	0.199*
INDEXF	0.054	1.000	0.788*	0.731*	0.326*	0.103
DISAREA	0.136	0.696*	1.000	0.694*	0.317*	0.293*
%DISAREA	0.143	0.780*	0.576*	1.000	0.284*	−0.236*
%DISPOINTS	0.253*	0.294*	0.168	0.263*	1.000	0.123
TOTAREA	0.007	−0.003	0.408*	−0.376*	−0.107	1.000

Correlations between traits of inoculation 1 are shown above the diagonal, whereas correlations of inoculation 2 are shown below the diagonal.

*Significantly different at p ≤ 0.05. INDEX0 (disease index based on a visual 1 to 9 rating scale taken 6 days after inoculation), INDEXF (disease index based on a visual 1 to 9 rating scale taken 14 days after inoculation), DISAREA (diseased leaf area measured in cm2), %DISAREA (percentage of diseased leaf area), %DISPOINTS (percentage of points showing symptoms), TOTAREA (total leaf area measured in cm^2^).

**Figure 1:**
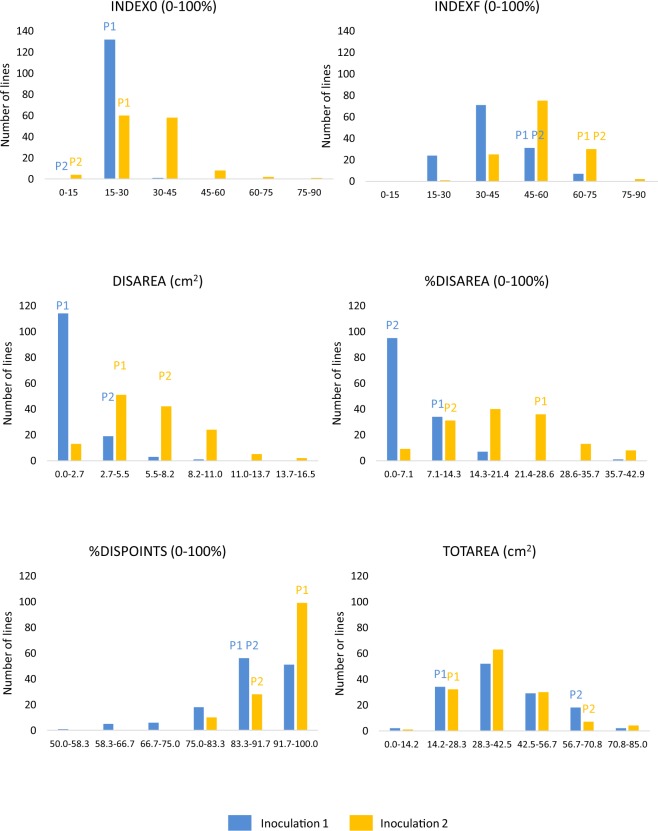
Distribution of resistant traits in the BolTBDH population in both inoculations. Classes where values of P1 (DH rapid cycling of Chinese kale TO1000DH3) and P2 (DH broccoli line ‘Early Big’) are included are shown.

There were significant differences between parents for %DISAREA and SCORE0 in the first inoculation. Generally speaking, parents were less damaged in the first inoculation than in the second one (Table 2). This is confirmed in the distribution of resistant traits through the whole set of lines of the BolTBDH population (Fig. 1). Although most individuals in the population are distributed inside the interval marked by parents, some of them showed transgressive distribution, being more resistant than parents. We can also highlight that almost the entire population shows symptoms at all points of inoculation.

**Table 2:** Means ± standard errors, minimum and maximum values of resistance traits and leaf area of P1 (DH rapid cycling of Chinese kale TO1000DH3) and P2 (DH broccoli line ‘Early Big’) in both inoculations.

Traits	P1-inoculation 1			P2-inoculation 1			P1-inoculation 2			P2-inoculation 2		
	Mean	Min	Max	Mean	Min	Max	Mean	Min	Max	Mean	Min	Max
INDEX0	20.00 ± 3.14	17.78	22.22	14.44 ± 1.57	13.36	15.56	19.44 ± 3.93	16.67	22.22	11.11 ± 0.00	11.11	11.11
INDEXF	51.67 ± 2.36	50.00	53.33	45.83 ± 1.96	44.44	47.22	61.94 ± 12.96	52.78	71.11	59.72 ± 9.82	52.78	66.67
DISAREA	1.98 ± 0.79	0.78	3.21	3.13 ± 1.82	0.92	6.20	3.68 ± 3.34	0.38	8.73	6.25 ± 7.19	0.45	19.62
%DISAREA	11.78 ± 5.98	4.20	22.89	5.02 ± 3.28	1.27	10.26	22.81 ± 26.93	1.70	90.08	8.75 ± 9.03	1.05	25.83
%DISPOINTS	91.67 ± 11.79	83.33	100.00	90.28 ± 1.96	88.89	91.67	95.83 ± 5.89	91.67	100.00	86.11 ± 3.93	83.33	88.89
TOTAREA	17.94 ± 4.25	12.18	24.12	66.33 ± 14.7	41.07	80.95	19.85 ± 9.07	7.99	32.67	60.18 ± 21.94	27.25	83.12

Nine and seven individuals were measured for P1 and P2, respectively.

INDEX0 (disease index based on a visual 1 to 9 rating scale taken 6 days after inoculation), INDEXF (disease index based on a visual 1 to 9 rating scale taken 14 days after inoculation), DISAREA (diseased leaf area measured in cm^2^), %DISAREA (percentage of diseased leaf area), %DISPOINTS (percentage of points showing symptoms), TOTAREA (total leaf area measured in cm^2^).

### QTL mapping

**Figure 2:**
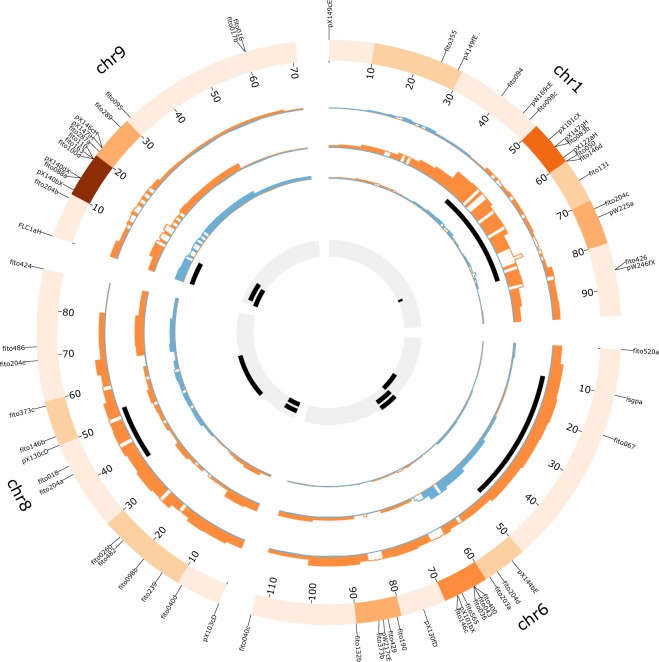
Circos graph of detected single-trait QTLs. Track I, represents the karyotype of *B*. *oleracea* showing markers used for QTL detection. The heatmap indicates marker density per 10 cM. Tracks from II to IV. Representation of detected QTLs per trait. Histogram represents the genetic contribution of each parental (orange: P1; blue: P2). The confidence intervals for detected QTLs are represented in black boxes. Track II: DISAREA. Track III: INDEX0. Track IV:%DISAREA. Track V represents QTLs of phenolic^15^ and GSLs^16^ content (black).

Three QTLs related to resistance of BolTBDH population to *Xcc* were found across three linkage groups in the first inoculation (Fig. 2, Table 3). Adjusted R^2^ varied between 7.89 for Xcc6.1 to 12.6% for Xcc1.1 (Table 3). Alleles of resistance were given by P1. To relate resistance to the area of the leaf QTLs of TOTAREA were also mapped (Table 3, Fig. 2). Three QTLs were localized in linkage groups 6, 8 and 9, explaining 25.8, 5.7 and 14.3% of variation, respectively. One QTL of resistance, Xcc9.1, was found in the second inoculation explaining 12.8% variation (Table 3, Fig. 2). Two QTLs of TOTAREA were detected in linkage groups 6 and 9, explaining 9.2 and 21.6% of variation, respectively. Alleles to increase resistance and leaf size were given by P2.

**Table 3:** Characteristics of single-trait QTLs for resistance traits and leaf size found in the BolTBDH mapping population inoculated with race 1 of *Xanthomonas campestris* pv. *campestris* (*Xcc*) in two consecutive inoculations.

QTL	Linkage group	Position (cM)	Trait	Profile LOD	LOD threshold	Additive effect	partR2	QTLs of resistance in other populations
**QTLs related to resistance**								
Inoculation 1								
Xcc1.1	1	76 (46–81)	INDEX0	3.91	2.24	0.898	12.66	BRQTL-C1_1, BRQTL-C1_2 (1)
Xcc6.1	6	23 (9–48)	DISAREA	2.70	2.16	0.389	7.89	BRQTL-C6 (1)
Xcc8.1	8	44 (37–53)	DISAREA	3.73	2.16	0.423	9.69	XccBo(Reiho)2 (2)
Inoculation 2								
Xcc9.1	9	2 (0–10)	%DISAREA	3.87	2.28	−3.310	12.76	
**QTLs related to leaf size**								
Inoculation 1								
Area6.1	6	38 (32–47)	TOTAREA	7.66	2.23	7.363	25.74	
Area8.1	8	41 (28–59)	TOTAREA	2.23	2.23	3.109	5.68	
Area9.1	9	9 (3–17)	TOTAREA	4.72	2.23	4.796	14.28	
Inoculation 2								
Area6.2	6	63 (53–73)	TOTAREA	3.14	2.29	3.751	9.17	
Area9.2	9	5 (0–15)	TOTAREA	6.01	2.29	6.528	21.57	

Shown are those overlapping QTLs of resistance to *Xcc* found in other mapping populations.

(1)^25^, (2)^26^. INDEX0 (disease index based on a visual 1 to 9 rating scale taken 6 days after inoculation), INDEXF (disease index based on a visual 1 to 9 rating scale taken 14 days after inoculation), DISAREA (diseased leaf area measured in cm^2^), %DISAREA (percentage of diseased leaf area), %DISPOINTS (percentage of points showing symptoms), TOTAREA (total leaf area measured in cm^2^).

We have found several coincidences of position of our single-trait QTLs and those found by other authors in *B*. *oleracea* based on the comparison of markers (Table 3). In the interval mapping of Xcc1.1^25^, found two QTLs BRQTL-C1_1 and BRQTL-C1_2. The same authors also found the QTL BRQTL-C6 in the confidence interval where Xcc6.1 is located. Marker OL12-DO5 is located in the confidence interval of Xcc8.1 and was also in the confidence interval of QTL XccBo(Reiho)2 in^26^.

To test the presence of QTLs which simultaneously affect resistance and leaf area a multi-QTL approach was carried out. Five multi-QTLs were detected across five linkage groups (Table 4). All of them were related to different resistance traits and also to leaf size. A new QTL in linkage group 5 was detected respect to single-trait analysis. In all multi-trait QTLs, alleles for increasing leaf size are given by P2. Alleles for increasing resistance measured as INDEX0 and %DISPOINTS are given by both parents in different QTLs, whereas alleles for increasing resistance measured as INDEXF and DISAREA are given by P1. Alleles for decreasing %DISAREA are given also by both parents.

**Table 4:** Significant QTLs from multi-trait analyses of resistant traits and leaf area in BolTBDH mapping population inoculated with race 1 of *Xanthomonas campestris* pv. *campestris* (*Xcc*).

QTL	Linkage group	Position	−LOG10(p)	Trait	Inoculation	QTL × trait additive effect	s.e.	Allele for increasing resistance	Allele for increasing leaf size	%Expl. Var.
MQ1.1	1	31.7	7.42	%DISPOINTS	1	0.247	0.092	P1		6.1
				INDEX0	1	0.384	0.087	P1		14.7
				TOTAREA	1	−0.268	0.071		P2	7.2
MQ5.1	5	64.1	5.50	%DISPOINTS	2	0.280	0.090	P2		7.8
				INDEX0	2	0.255	0.089	P2		6.5
				INEDXF	1	0.230	0.087	P1		5.3
				TOTAREA	1	−0.200	0.069		P2	4.0
MQ6.1	6	45.3	9.60	DISAREA	1	0.235	0.087	P1		5.5
				TOTAREA	1	−0.467	0.071		P2	21.8
				TOTAREA	2	−0.330	0.082		P2	10.9
MQ8.1	8	50.2	5.47	%DISAREA	1	0.242	0.089	P1		5.9
				DISAREA	1	0.301	0.082	P1		9.0
				INDEX0	2	−0.191	0.087	P2		3.7
				INEDXF	1	0.200	0.084	P1		4.0
				TOTAREA	1	−0.211	0.067		P2	4.5
				TOTAREA	2	−0.154	0.077		P2	2.4
MQ9.1	9	14.5	9.80	%DISAREA	2	−0.299	0.089	P2		9.0
				DISAREA	1	0.167	0.084	P1		2.8
				INDEX0	1	0.219	0.085	P1		4.8
				INEDXF	1	0.263	0.087	P1		6.9
				TOTAREA	1	−0.384	0.069		P2	14.8
				TOTAREA	2	−0.412	0.079		P2	17.0

The percentage of variability explained is shown for every trait that had a significant (p ≤ 0.05) interaction with the QTL.

INDEX0 (disease index based on a visual 1 to 9 rating scale taken 6 days after inoculation), INDEXF (disease index based on a visual 1 to 9 rating scale taken 14 days after inoculation), DISAREA (diseased leaf area measured in cm^2^), %DISAREA (percentage of diseased leaf area), %DISPOINTS (percentage of points showing symptoms), TOTAREA (total leaf area measured in cm^2^).

**Figure 3:**
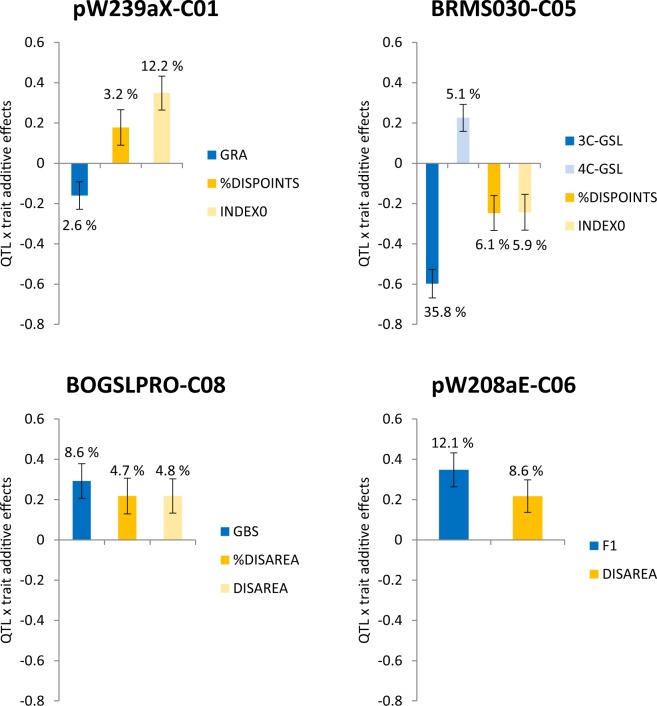
Bar plots showing the QTL × additive effect ± standard errors of four multi-trait QTLs controlling variation for metabolite content and resistance. Only traits that had a significant interaction (p ≤ 0.05) with the QTL are shown. The percentage of variability explained by trait is shown on each bar.

To carry on multi-trait QTL analysis to find QTLs related to resistance and content of phenolics and GSLs, we took into account previously mapped consensus QTLs for content of cited metabolites in leaves, which were coincident in position with multi-trait QTLs for resistance (Table 5). Three QTLs related to GSL content^16^ and two related to phenolic content^15^ were located in the same confidence interval as several multi-trait QTLs (Table 5). For those areas we have tested the presence of multi-trait QTL controlling variation for metabolites content and resistance to *Xcc*. Four multi-trait QTLs were found across four linkage groups (Fig. 3). In pW239aX marker, genotypes carrying the alleles to reduce INDEX0 and %DISPOINTS have also smaller quantity of GRA than genotypes carrying the allele to increase it (Fig. 3). The mQTL5.1 (Table 5) was described as locus GSL-PRO^16^ which is involved in the elongation of the aliphatic GSL side chain. Allele GSL-PRO+, present in P1, induce the production of 3C-GSLs (SIN, GIB), whereas allele from P2 induces the presence of 4C-GSLs (GRA, GNA and PRO). Resistance expressed as SCORE0 and %DISPOINTS is related to the presence of 4C-GSLs in marker BRMS030 (Fig. 3). mQTL8.1^16^ controls the amount of the indolic GSLs: GBS, NEOGBS and OHGBS in leaves (Table 5). A multi-trait QTL was found in marker BOGSLPRO controlling the amount of GBS, %DISAREA and DISAREA. Both resistant traits increase when GBS increase (Fig. 3). Regarding coincidences of QTLs of resistance with QTLs for phenolic compounds^15^, QTL6.1 controls the amount of flavonoids F1 and F7. In marker pW208aE, genotypes carrying the allele for increasing the amount of F1 are more susceptible (Fig. 3). There no was multi-trait QTL for 3pCoQAc amount and resistance.

**Table 5:** Description of consensus QTLs for phenolic content and GSLs mapped in the population BolTBDH by^15^ and^16^, respectively.

QTL	Linkage group	Trait	High value allele	Position (cM)	Confidence interval
**Consensus QTLs of GSLs content**					
mQTL1.1	1	GRA	P2	73	72–74
mQTL5.1	5	3C-GSLs	P1	70	68–72
		4C-GSLs	P2		
mQTL8.1	8	GBS	P2	46	42–50
		NEOGBS	P2		
		OHGBS	P1		
**Consensus QTLs of phenolic content**					
QTL6.1	6	F1	P2	54	45–58
		F7	P2		
QTL8.1	8	3pCoQAc	P1	3	0–10

GRA: glucoraphanin; GNA: gluconapin; PRO: Progoitrin; GBS: glucobrassicin; NEOGBS: neoglucobrassicin; OHGBS: hydroxyglucobrassicin; 3C-GSLs: sum of GRA, GNA and PRO; 4C-GSL: sum of sinigrin (SIN) and glucoiberin (GIB); F1: kaempferol-3-O-(methoxycaffeoyl)diglucoside-7-O-glucoside; F7: kaempferol-3-O-(caffeoyl, sinapoyl)triglucoside; 3pCoQAc: 3-*p*-coumaroyl quinic acid.

### Candidate genes underlying variation for multi-trait QTLs

Confidence intervals of multi-trait QTLs corresponded to genomic regions inside chromosomes 1, 3, 4 and 5 of *A*. *thaliana* (Table 6). MQ5.1 and MQ8.1 showed an overlapping syntenic region in chromosome 1 of *A*. *thaliana*, whereas the rest of syntenic regions were independent. GO analysis of Biological Process showed that genes present in all the genomic regions of *A*. *thaliana* related to QTLs of resistance were significantly enriched in one or more GO terms (Table S1). GO: 0010876 (lipid localization) was significant in MQ1.1 (in both regions of chromosomes 3 and 4) and in MQ5.1. Within this category, fifteen genes related to lipid transfer proteins (LTPs) were found in both QTLs (Table S2). GO: 0042742 (defense response to bacterium) was significant in MQ1.1. Within this category we found genes related to different stages of plant defense against bacteria (Table S2). GO: 0009809 (lignin metabolic process) was significant in MQ8.1 and GO: 0034637 (cellular carbohydrate biosynthetic process) was significant in MQ5.1 and MQ8.1. Genes related to phenolic compounds and GSLs biosynthesis were found within these categories (Table S2).

**Table 6:** Correspondence between genomic regions of *Brassica oleracea* within confidence interval of multi-trait QTLs and *Arabidopsis thaliana* genome.

*Brassica oleracea*			*Arabidopsis thaliana*			
QTL	Linkage group	Markers	Chromosome	Coordenates (bp)		Number of genes
MQ1.1	1	pW249dE-fito083b	3	1,565,867	16,873,329	4019
		pX122aH-pW239bX	4	11,600,491	15,766,114	1530
MQ5.1	5	fito156a-pX126bX	1	3,530,200	8,997,298	2050
MQ6.1	6	pW208aE-pX144bE	1	28,255,695	29,491,879	430
MQ8.1	8	fito098b-pW231aX	1	2,787,156	6,785,496	2315
		pW130aE-pX130cD	3	19,920,323	22,757,895	1018
MQ9.1	9	FLC1aH-pW212aE	5	3,173,498	6,427,450	1200

## Discussion

Resistance to *Xcc* was measured according five traits in the BolTBDH mapping population. INDEX0 was taken in the initial days after the inoculation, whereas the rest of traits (INDEXF, DISAREA, %DISAREA and %DISPOINTS) were recorded 14 days after inoculation. INDEX0 and %DISPOINTS did not show very high correlations with the rest of resistant traits, meaning that they may measure different aspects of plant resistance. INDEX0 would be related to plant mechanisms of resistance at initial stages of the invasion by *Xcc*, whereas %DISPOINTS would be related to the success of infection. The rest of traits are more related to the spread of the pathogen after initial infection. There were significant differences between parents only for traits %DISAREA and INDEX0 in the first inoculation, in which P2 showed less damage than P1. P2 has bigger leaves than P1. Therefore, when only the area of the lesion is considered, without relativizing that to the total leaf, P1 is more resistant than P2 (lower DISAREA), but no in a significant way.

Resistance traits and leaf area showed continuous distributions in both inoculations. Plants in the second inoculation were more damaged than in the first one, meaning that plants were not fully recovered after elimination of the damaged leaves in the first one. Independent single QTL analysis was done for resistant traits on each inoculation. We also performed QTL analysis for TOTAREA to relate the variation of this trait to variation of resistance.

### QTL mapping

Four single-trait QTLs related to resistance to race 1 of *Xcc* have been found in the BolTBDH mapping population. Therefore, resistance is quantitative and under polygenic control, confirming results found by other authors^25–29^. Three QTLs are probably related to other resistance QTLs found previously^25–29^, whereas QTL Xcc9.1 represent novel variation.

Few qualitative disease resistance genes against xylem-colonizing bacterial pathogens have been developed or identified so far^7^. *Xcc* infects plants mainly through hydathodes and should colonize epitheme. This pathogen kills host cells probably by degrading cell walls. Epitheme cells may be destroyed so rapidly that any R gene expression may be ineffective^7^. Although qualitative resistance has not been found in our work, previous studies showed that, in some cases, QTLs may be R genes that have lost their qualitative feature and adopted new, intermediate resistance phenotypes^30^. In *Arabidopsis* the immune receptor pair of TIR-NBS-LRR proteins conferring resistance to *P*. *syringe* and *Ralstonia Solanacearum* confers partial resistance to a single race of *Xcc*^31^. Xcc1.1 found in our work is probably related to BRQTL-C1_1 and BRQTL-C1_2 of^25^, Xcc6.1 is probably related to BRQTL-C6 of^25^. Following^25^ variability in NBS-LRR type R genes found in the confidence interval of BRQTL-C1_1, BRQTL-C1_2 and BRQTL-C6 could be responsible for resistance to *Xcc*.

Five QTLs were found in multi-trait QTL analysis. Basically, MQ1.1 and MQ5.1 control resistance in the initial stages of invasion and success of infection, measured as and INDEX0 and %DISPOINTS. Alleles for increasing resistance in MQ1.1 are given by P1, whereas P2 gives resistant alleles in MQ5.1. In this last QTL and once invasion takes place, spread of the disease is controlled by P1. Alleles to decrease INDEX0 are given by different parent than those to decrease traits related to the spread of the disease in MQ8.1 and MQ9.1. Therefore, it seems that there are two different mechanisms of resistance controlled by the same multi-trait QTLs. In MQ8.1 and MQ9.1 relationship among %DISPOINTS and SCORE0 with TOTAREA was not consistent across multi-QTLs, indicating a complex relationship among these traits.

Even that P1 has smaller leaves it is able to reduce the lesion size in MQ6.1 and MQ8.1. From this point of view, cited QTLs would be interesting to manage in a breeding program to obtain crops with small diseased area if the end-use of the crop is for their leaves (such as kale or cabbage). When the disease area is relativized to the total area of the leaf, individuals carrying allele from P2 in MQ9.1 perform better than those carrying allele from P1. Plants with bigger leaves could have more chances to survive to *Xcc* invasion because they can tolerate better damage caused by the pathogen. Assuming that the disease advances at the same speed in all the leaves, pathogen will invade a leaf with a smaller surface in less time than a leaf with a larger one. Plants with bigger leaves could stop the expansion of chlorosis while maintaining part of the leaf photosynthetically active. The distance between the inoculation points could also influence the size of chlorotic spots. Distance between inoculation points is lower in plants carrying allele from P1 than those carrying allele from P2. Therefore, in a small leaf, the chlorosis spots will be grouped forming a larger spot in less time than in a larger leaf. On the contrary, in a large leaf, the diseased areas remain isolated for longer and that time can be determinant for the plant to react to the pathogen, leaving only some areas of the leaf dead and keeping the rest of the leaf photosynthetically active. In the particular case of P2, a broccoli type inbred line, the genetic control of tolerance to leaf invasion by *Xcc* determined by QTL MQ9.1could be interesting to manage, since plants can keep photosynthetically active areas of the leaves after invasion. This could allow the plant to send resources to form the florets of broccoli. This relationship between tolerance and size of the leaves was not previously described in the interaction *Brassica-Xcc*. In an early work, searching for QTLs of resistance to *Xcc* in *B*. *oleracea*^27^, also mapped QTLs related to leave size, but no co-location was detected. However, resistance to *Xcc* in *B*. *oleracea* was previously related to the ability of the plant to keep sufficient photosynthesis metabolism activity to provide energy supplies necessary for defence^32–34^.

Quantitative resistance can also be associated to the production of secondary metabolites. Genes encoding cytochrome P450 were highly induced in a resistant line of cabbage after inoculation with *Xcc*^34^. This superfamily participates in the phenylpropanoid pathway, related to the biosynthesis of phenolic compounds (hydroxycinnamic acids, lignins, flavonoids). Phenolic compounds have antimicrobial activity *in vitro* against bacterial pathogens of plants^13,14^. Spread of the pathogen could also be stopped by via peroxidase-mediated increases in lignifications in *B*. *oleracea*^33^. Contrarily, in this work we have found that increasing the amount of flavonoids F1 does not results in and reduction in DISAREA.

The relationship between the content of GSLs and resistance to *Xcc* was dependent on the type of GSL. GSL-PRO controls the side-chain elongation of aliphatic GSLs, from 3 C to 4 C. Increasing the amount of 4C-GSL increases resistance expressed as INDEX0 and %DISPOINTS, and therefore, inhibits initial stages of the infection caused by *Xcc*. The 4C-GSLs present in the leaves of the mapping population are GRA, GNA and PRO, whereas 3C-GSLs are SIN and GIB. However, increasing the amount of GRA is related to the increase in INDEX0 and %DISPOINTS. The *in vivo* antibiotic effect of GRA on bacteria is dependent on the pathogen species. The suppression of GRA in Arabidopsis does not have effect on resistance against *E*. *carotovora* and *P*. *syringae*^35,36^. The effect of GSL-PRO in INDEX0 and %DISPOINTS would be more related to the relative proportion of 4C-GSL *vs* 3C-GSL rather than to the concentration of individual GSLs. Coincidently^37^, proved that *in vitro* activity of aliphatic isothiocyanates (hydrolytic products from GSLs) against bacteria *E*. *carotovora* was general intensive when the chain length was increased. Finally, increment in GBS leads to increment in DISAREA and %DISAREA.

### Candidate genes underlying variation for multi-trait QTLs

To find possible candidate genes accounting for *Xcc* resistance, a GO analysis with genes present in genomic areas of *A*. *thaliana* syntenic to those of multi-trait QTLs of resistance was done. Interestingly, we found several GO categories related directly or indirectly to disease resistance. Plants protect themselves from microbial pathogens through the recognition of microbe-associated molecular patterns (PAMPs). The perception often triggers a series of defence responses, including the generation of ROS, induction of mitogen-activated protein kinases (MAPKs), pathogenesis-related (PR) gene expression, callose deposition at the cell wall, camalexin accumulation and, in some cases, a hypersensitive response.

Within GO significant categories, we have found genes involved in different aspects of plant immunity. RPS2 is a NBS-LRR disease resistance gene and WIN2 is a protein serine/threonine phosphatase, which are involved in the recognition avirulence proteins from *P. syringae*^38,39^. Responses to pathogen attack require large-scale transcriptional reprogramming, including transcription factors such as WRKY genes^40^. WRKY53 is a positive regulator of basal resistance, whereas WRKY11 and WRKY18 act as negative regulators of defense signaling^40^. Proteins CRK13 and CRK11 are cysteine-rich receptor-like kinases (CRKs). The induction of CRKs during immune responses suggests that they cooperate to enhance defense signaling^41^. A subset of CRKs is strongly induced in response to pathogens and PAMP treatment. Upregulation of CRK13 leads to hypersensitive response-associated cell death^42^.

LTPs reversibly bind lipids and deliver them to their destination. Among a plethora of functions, these proteins are components of plant innate immunity. Their toxicity seems to be related to their ability to promote membrane permeabilization in pathogen, but no in host cells^43^. Many isoforms are induced upon pathogen challenge, thus, leading to the definition of LTPs as a family of PR proteins (PR14)^44^. GSH1 and VTC2 are related to the biosynthesis of glutathione and ascorbate, respectively. Both molecules are involved in the redox homeostasis of plant cells which can be broken upon pathogen attack. We have also found genes related to lignin and GSLs biosynthesis. In this last case, MYB51 is a transcription factor which induces the synthesis of indolic GSLs, and could be responsible for co-variation between GBS content and %DISAREA and DISAREA in linkage group 8.

## Conclusions

Four QTLs of resistance of *B*. *oleracea* to race 1 of *Xcc* were found in the BolTBDH mapping population. Several traits related to resistance were analyzed, including one related to initial stages of the infection, another to the success of infection and three related to the spread of the disease. After performing multi-trait QTL, we concluded that spread of *Xcc* is related to the size of the leaf. Genotypes from the mapping population would follow two different strategies to cope with the spread of the disease. Those with alleles from P1 have a reduced lesion size, whereas genotypes with alleles from P2 maintain more area of the leaf with photosynthetic activity, being in this way more tolerant to *Xcc* invasion. Mechanisms underlying variation for resistance may be related to different aspects of plant immunity, including the synthesis of GSLs and phenolics.

## Supplementary information

Table S1 and S2
