# Supplementary material for: Dissecting quantitative resistance to Xanthomonas campestris pv. campestris in leaves of Brassica oleracea by QTL analysis
Source: Sci Rep. 2019 Feb 14;9:2015. doi: 10.1038/s41598-019-38527-5 (PMC6376059; doi:10.1038/s41598-019-38527-5)
Supplement: Supplementary file 1 — Table S1 and S2 [file 41598_2019_38527_MOESM1_ESM.pdf]

Dissecting quantitative resistance to *Xanthomonas campestris* pv. *campestris* in leaves of *Brassica oleracea* by QTL analysis

Laura Iglesias-Bernabé [1], Pari Madloo[2], Víctor Manuel Rodríguez[1], Marta Francisco[1], Pilar Soengas[1*]

[1] Group of Genetics, Breeding and Biochemistry of Brassicas, Misión Biológica de Galicia (MBG-CSIC), Pontevedra, Spain

[2]University of Santiago de Compostela, Spain

Laura Iglesias Bernabé: lauib@uvigo.es

Pari Madloo: paribrokanloui.Madloo@rai.usc.es

Víctor Manuel Rodríguez: vmrodriguez@mbg.csic.es

Marta Francisco: mfrancisco@mbg.csic.es

*Corresponding author: Pilar Soengas: psoengas@mbg.csic.es

TLF:+34986854800

Table S1. List of significant enriched GO terms related to Biological Process in *Arabidopsis thaliana* regions related to QTLs of resistance in *Brassica oleracea*

**MQ1.1**	**Arabidopsis chr3:1,565,867-16,873,329**				
**GO term**	**Description**	**Number in input list**	**Number in BG/Ref**	**p-value**	**FDR**
GO:0010876	lipid localization	17	24	1.50E-08	5.40E-05
**MQ1.1**	**Arabidopsis chr4: 11,600,491-15,766,114**				
GO:0010876	lipid localization	23	24	1.40E-20	3.70E-17
GO:0046087	cytidine metabolic process	8	9	7.90E-08	3.80E-05
GO:0046131	pyrimidine ribonucleoside metabolic process	9	14	8.50E-08	3.80E-05
GO:0006216	cytidine catabolic process	8	9	7.90E-08	3.80E-05
GO:0033036	macromolecule localization	47	462	3.00E-08	3.80E-05
GO:0009972	cytidine deamination	8	9	7.90E-08	3.80E-05
GO:0046135	pyrimidine nucleoside catabolic process	8	10	1.40E-07	4.20E-05
GO:0046133	pyrimidine ribonucleoside catabolic process	8	10	1.40E-07	4.20E-05
GO:0006213	pyrimidine nucleoside metabolic process	9	15	1.30E-07	4.20E-05
GO:0009164	nucleoside catabolic process	8	11	2.30E-07	5.70E-05
GO:0042454	ribonucleoside catabolic process	8	11	2.30E-07	5.70E-05
GO:0034655	nucleobase, nucleoside, nucleotide and nucleic acid catabolic process	8	12	3.70E-07	7.80E-05
GO:0034656	nucleobase, nucleoside and nucleotide catabolic process	8	12	3.70E-07	7.80E-05
GO:0008152	metabolic process	506	10614	1.60E-06	0.00031
GO:0034641	cellular nitrogen compound metabolic process	45	506	1.80E-06	0.00033
GO:0006869	lipid transport	22	163	2.60E-06	0.00044
GO:0015908	fatty acid transport	6	7	4.20E-06	0.00067

GO:0046483	heterocycle metabolic process	41	460	4.80E-06	0.00073
GO:0009987	cellular process	546	11684	5.10E-06	0.00073
GO:0015718	monocarboxylic acid transport	6	8	7.10E-06	0.00096
GO:0044237	cellular metabolic process	419	8722	1.10E-05	0.0014
GO:0009116	nucleoside metabolic process	11	50	1.70E-05	0.0022
GO:0009791	post-embryonic development	53	705	2.10E-05	0.0025
GO:0000165	MAPKKK cascade	6	11	2.60E-05	0.003
GO:0009119	ribonucleoside metabolic process	9	35	3.60E-05	0.0039
GO:0044238	primary metabolic process	424	8995	5.30E-05	0.0055
GO:0046700	heterocycle catabolic process	9	38	6.20E-05	0.0062
GO:0055086	nucleobase, nucleoside and nucleotide metabolic process	23	221	7.00E-05	0.0068
GO:0042742	defense response to bacterium	20	177	7.30E-05	0.0069
GO:0002237	response to molecule of bacterial origin	6	15	0.0001	0.0092
GO:0046148	pigment biosynthetic process	15	112	0.00011	0.0093
GO:0042538	hyperosmotic salinity response	10	54	0.00015	0.013
GO:0042440	pigment metabolic process	16	134	0.00021	0.017
GO:0015995	chlorophyll biosynthetic process	9	47	0.00025	0.02
GO:0010078	maintenance of root meristem identity	5	11	0.00025	0.02
GO:0018130	heterocycle biosynthetic process	15	123	0.00027	0.02
GO:0032318	regulation of Ras GTPase activity	8	37	0.00027	0.02
GO:0042221	response to chemical stimulus	116	2085	0.00029	0.02
GO:0007243	protein kinase cascade	6	19	0.00029	0.02
GO:0051179	localization	108	1922	0.00033	0.023
GO:0043087	regulation of GTPase activity	8	39	0.00037	0.025
GO:0006796	phosphate metabolic process	72	1178	0.00039	0.025
GO:0006793	phosphorus metabolic process	72	1179	0.0004	0.025
GO:0046578	regulation of Ras protein signal transduction	8	41	0.0005	0.027
GO:0051336	regulation of hydrolase activity	8	41	0.0005	0.027

GO:0006972	hyperosmotic response	10	64	0.0005	0.027
GO:0051056	regulation of small GTPase mediated signal transduction	8	41	0.0005	0.027
GO:0007265	Ras protein signal transduction	8	41	0.0005	0.027
GO:0016192	vesicle-mediated transport	24	272	0.00048	0.027
GO:0044248	cellular catabolic process	50	746	0.00046	0.027
GO:0051186	cofactor metabolic process	26	308	0.00053	0.028
GO:0006810	transport	103	1846	0.00057	0.03
GO:0015994	chlorophyll metabolic process	10	66	0.00062	0.031
GO:0051234	establishment of localization	103	1851	0.00062	0.031
GO:0016310	phosphorylation	66	1079	0.00066	0.033
GO:0010073	meristem maintenance	10	68	0.00076	0.037
GO:0007264	small GTPase mediated signal transduction	8	46	0.00097	0.046
**MQ5.1**	**Arabidopsis chr1: 3,530,200-8,997,298**				
GO:0034637	cellular carbohydrate biosynthetic process	31	177	3.20E-08	0.0001
GO:0044262	cellular carbohydrate metabolic process	48	417	9.90E-07	0.0016
GO:0009880	embryonic pattern specification	8	12	2.30E-06	0.0019
GO:0009791	post-embryonic development	68	705	2.30E-06	0.0019
GO:0016051	carbohydrate biosynthetic process	33	277	2.50E-05	0.016
GO:0007389	pattern specification process	22	150	3.60E-05	0.019
GO:0046493	lipid A metabolic process	6	10	7.00E-05	0.028
GO:0009245	lipid A biosynthetic process	6	10	7.00E-05	0.028
GO:0008653	lipopolysaccharide metabolic process	6	11	0.0001	0.033
GO:0009103	lipopolysaccharide biosynthetic process	6	11	0.0001	0.033
GO:0010876	lipid localization	8	24	0.00012	0.033
GO:0008654	phospholipid biosynthetic process	15	88	0.00014	0.037
**MQ6.1**	**Arabidopsis chr1: 28,255,695-29,491,879**				

GO:0019953	sexual reproduction	7	80	1.10E-05	0.0065
GO:0009404	toxin metabolic process	5	53	0.00015	0.029
GO:0009407	toxin catabolic process	5	53	0.00015	0.029
**MQ8.1**	**Arabidopsis chr1: 2,787,156-6,785,496**				
GO:0034637	cellular carbohydrate biosynthetic process	22	177	6.80E-06	0.007
GO:0044262	cellular carbohydrate metabolic process	37	417	1.10E-05	0.007
GO:0009809	lignin biosynthetic process	11	42	3.70E-06	0.007
GO:0009808	lignin metabolic process	11	47	9.20E-06	0.007
GO:0008037	cell recognition	8	32	0.0001	0.044
GO:0048544	recognition of pollen	8	32	0.0001	0.044
**MQ8.1**	**Arabidopsis chr3: 19,920,323-22,757,895**				
GO:0034641	cellular nitrogen compound metabolic process	39	506	2.10E-08	3.40E-05
GO:0000096	sulfur amino acid metabolic process	15	84	4.60E-08	3.90E-05
GO:0009066	aspartate family amino acid metabolic process	14	90	5.80E-07	0.00032
GO:0044106	cellular amine metabolic process	32	438	1.10E-06	0.00046
GO:0006520	cellular amino acid metabolic process	31	430	2.10E-06	0.0007
GO:0006897	endocytosis	7	20	4.60E-06	0.0011
GO:0010324	membrane invagination	7	20	4.60E-06	0.0011
GO:0008652	cellular amino acid biosynthetic process	19	202	6.60E-06	0.0014
GO:0006555	methionine metabolic process	10	56	8.10E-06	0.0015
GO:0009821	alkaloid biosynthetic process	6	16	1.60E-05	0.0028
GO:0009768	photosynthesis, light harvesting in photosystem I	5	9	2.00E-05	0.003
GO:0009308	amine metabolic process	32	521	3.10E-05	0.0043
GO:0009309	amine biosynthetic process	19	229	3.40E-05	0.0043
GO:0044271	cellular nitrogen compound biosynthetic process	26	394	5.50E-05	0.0066
GO:0000097	sulfur amino acid biosynthetic process	8	49	0.00011	0.013

GO:0009069	serine family amino acid metabolic process	8	54	0.00021	0.022
GO:0006519	cellular amino acid and derivative metabolic process	35	682	0.00037	0.036
GO:0009067	aspartate family amino acid biosynthetic process	7	45	0.00039	0.037
GO:0006790	sulfur metabolic process	16	220	0.00054	0.047
**MQ9.1**	**Arabidopsis chr5: 173,498-6,427,450**				
GO:0000904	cell morphogenesis involved in differentiation	12	63	2.70E-06	0.0051

Table S2. Possible candidate genes underlying variation for multi-trait QTLs for resistance to *Xanthomonas campestris pv. campestris*

AGI number	Name	Description	multi-trait QTL
**GO:0010876**	**lipid localization**		
AT3G22620		Lipid transfer protein	MQ1.1, MQ5.1
AT3G22580		Lipid transfer protein	MQ1.1, MQ5.1
AT3G18280		Lipid transfer protein	MQ1.1, MQ5.1
AT3G43720		Lipid transfer protein	MQ1.1, MQ5.1
AT3G22600		Lipid transfer protein	MQ1.1, MQ5.1
AT3G43720		Lipid transfer protein	MQ1.1, MQ5.1
AT4G22520		Lipid transfer protein	MQ1.1, MQ5.1
AT4G22460		Lipid transfer protein	MQ1.1, MQ5.1
AT4G22610		Lipid transfer protein	MQ1.1, MQ5.1
AT4G22490		Lipid transfer protein	MQ1.1, MQ5.1
AT4G30880		Lipid transfer protein	MQ1.1, MQ5.1
AT4G22485		Lipid transfer protein	MQ1.1, MQ5.1
AT4G22470		Lipid transfer protein	MQ1.1, MQ5.1
AT4G22630		Lipid transfer protein	MQ1.1, MQ5.1
AT1G18280		Lipid transfer protein	MQ1.1, MQ5.1
**GO:0042742**	**defense response to bacterium**		
AT4G23670		Major latex protein-related	MQ1.1
AT4G31800	WRKY18	Transcription factor	MQ1.1
AT4G31550	WRKY11	Transcription factor	MQ1.1
AT4G23810	WRKY53	Transcription factor	MQ1.1
AT4G23210	CRK13	Protein kinase family protein	MQ1.1

AT4G23190	CRK11	Protein kinase family protein	MQ1.1
AT4G23100	GSH1	Glutamate-cysteine ligase	MQ1.1
AT4G26850	VTC2	GDP-L-galactose phosphorylase 1	MQ1.1
AT4G26090	RPS2	NBS-LRR disease resistance gene	MQ1.1
AT4G32260		ATP synthase	MQ1.1
AT4G31500	CYP83B1	Cytochrome P450 Monooxygenase	MQ1.1
AT4G31750	WIN2	Protein serine/threonine phosphatase	MQ1.1
AT4G26070	MEK1	Map kinase	MQ1.1
**GO: 0034637**	**cellular carbohydrate biosynthetic process**		
AT1G16400	CYP79F2	Oxidoreductase, cytochrome P450 family	MQ5.1, MQ8.1
AT1G16410	CYP79F1	Oxidoreductase, cytochrome P450 family	MQ5.1, MQ8.1
AT1G18570	MYB51	R2R3-MYB transcription family	MQ5.1, MQ8.1
AT1G18590	SOT17	desulfoglucosinolate sulfotransferase	MQ5.1, MQ8.1
AT1G24100	UGT74B1	UDP-glucosyl transferase	MQ5.1, MQ8.1
AT1G12140	FMO GS-OX5	Flavin-monooxygenase	MQ5.1, MQ8.1
**GO: 0009809**	**lignin metabolic process**		
AT1G09510		Cinnamyl-alcohol dehydrogenase family	MQ8.1
AT1G09490		Cinnamyl-alcohol dehydrogenase family	MQ8.1
AT1G09500		Cinnamyl-alcohol dehydrogenase family	MQ8.1
AT1G09480		Cinnamyl-alcohol dehydrogenase family	MQ8.1
AT1G12840	DET3	Proton-transporting ATPase	MQ8.1
AT1G15950	CCR1	Cinnamoyl CoA reductase	MQ8.1
AT1G16490	MYB58	R2R3-MYB transcription family	MQ8.1
